# COVID-19 Posttraumatic Stress Disorder and Mental Health among Spanish Adolescents: SESSAMO Project

**DOI:** 10.3390/jcm13113114

**Published:** 2024-05-26

**Authors:** Nora Yárnoz-Goñi, Adriana Goñi-Sarriés, Azucena Díez-Suárez, Guillermo Pírez, Leticia Morata-Sampaio, Almudena Sánchez-Villegas

**Affiliations:** 1 Hospital Nuestra Señora de Gracia, Unidad de Trastornos de la Conducta Alimentaria, Servicio Aragonés de Salud, 50004 Zaragoza, Spain; nyarnozg@salud.aragon.es; 2 Red de Salud Mental de Navarra, Servicio Navarro de Salud-Osasunbidea, 31003 Pamplona, Spain; agonisar@navarra.es; 3 Instituto de Investigación Sanitaria de Navarra, IdiSNA, 31008 Pamplona, Spain; adiezs@unav.es; 4 Unidad de Psiquiatría Infantil y Adolescente, Departamento de Psiquiatría y Psicología Médica, Clínica Universidad de Navarra, 31008 Pamplona, Spain; 5 Complejo Hospitalario Universitario Insular-Materno Infantil, Servicio Canario de la Salud, 35016 Las Palmas de Gran Canaria, Spain; gpirmor@gobiernodecanarias.org; 6 Departamento de Psicología y Sociología, Universidad de Las Palmas de Gran Canaria, 35017 Las Palmas de Gran Canaria, Spain; leticia.morata@ulpgc.es; 7 Institute for Innovation & Sustainable Development in Food Chain, ISFOOD, Universidad Pública de Navarra (UPNA), 31006 Pamplona, Spain; 8 Biomedical Research Center Network on Physiopathology of Obesity and Nutrition (CIBEROBN), Institute of Health Carlos III, 28029 Madrid, Spain

**Keywords:** adolescents, COVID-19, mental health, posttraumatic stress

## Abstract

**Background**: Child and adolescent mental health problems have increased after the COVID-19 pandemic. The objective of this study was to establish the association of the presence and intensity of posttraumatic stress due to COVID-19 with the presence of (1) self-harm and suicide risk, (2) depressive and anxious symptoms, (3) eating disorders and (4) problematic Internet and video game use. **Methods**: A cross-sectional analysis was performed on a sample of second–fourth grade secondary school students (14 to 16 years old) from Navarra and the Canary Islands recruited at the SESSAMO project. Validated questionnaires were used to assess the intensity of posttraumatic stress due to COVID-19, risk of suicide and presence of self-harm, symptoms of mental disorder and problematic use of the Internet and video games. **Results**: Out of 1423 participants analyzed, those with the highest level of posttraumatic stress showed a significant increase in the risk of suicide (OR = 5.18; 95% CI = 2.96–9.05) and in the presence of eating disorder symptoms (OR = 3.93; 95% CI = 2.21–7.00), and higher anxiety and depression scores (b coefficient for anxiety = 11.1; CI = 9.7–12.5; for depression = 13.0; CI = 11.5–14.5) as compared to those with the lowest level. Participants with a high level of posttraumatic stress were almost 10 times more likely to present problematic video game use (OR = 9.49; 95% CI = 3.13–28.82). **Conclusions**: Years after the pandemic, posttraumatic stress derived from it continues to impact the mental health of adolescents. Further long-term research is needed, as well as close follow-up and intervention in this population.

## 1. Introduction

According to the World Health Organization (WHO), approximately 760 million people were infected worldwide by COVID-19 in 2023 [1]. The measures adopted during the pandemic in 2020, such as quarantine, travelling limitations, or closure of schools and companies, represented a turning point for the entire society. Subsequently, several studies have revealed mental health problems in the global population [2], with a 20% increase in mental disorders, especially in women and young people [3]. In 2021, the US Center of Disease Control conducted a population survey and highlighted that 37% of high school students had experienced mental health problems during the pandemic [4].

In the early stages, there were studies that showed high levels of depression and stress in adolescents, mainly attributed to social distancing, school closures and home isolation [5]. More precise estimates indicate a prevalence of anxiety of about 20.5–26%, and of depression of about 22.6–29% [6,7]. Other studies have compared the level of anxiety in the pre-pandemic period with that of the pandemic, finding higher levels, up to double the pre-pandemic estimates [8]. In studies carried out before the pandemic with Spanish adolescents in educational settings, it has been noted that 11.8% reported anxiety problems and 11.6% reported depression [9]. A recent meta-analysis reveals an increase in anxiety and depression symptoms after the pandemic, reaching 31% in both cases [10]. On the other hand, up to 48% of children and adolescents who had been isolated during this period met clinical criteria for posttraumatic stress disorder (PTSD) [7], defined as anxiety disorder that arises after having lived through a severely distressing event like COVID-19. The symptoms include arousal, avoidance and intrusive thoughts and feelings [11]. This usually leads to interference in psychological and social functioning while negatively impacting quality of life [12,13].

Suicidal behavior in adolescents is a public health problem due to its high prevalence and the personal, family and social consequences that entail. The prevalence of suicidal ideation increased by 17.5% among adolescents aged 16 to 18 years, compared to 6% in pre-pandemic estimates [14]. The review by Samji et al. shows a prevalence of suicidal ideation of about 6–37% and non-suicidal self-harm between 32% and 42% during that period [6]. There was an increase in suicide attempts with a relative risk of 1.22, especially in adolescents of 16–17 years of age [15], with a prevalence as high as 9% [5]. In 2022, suicide was the main cause of unnatural death among Spanish adolescents and young people [16].

The adolescent population has greater vulnerability, especially those who already had mental disorders prior to the COVID-19 crisis [17,18]. There has been a worsening of serious mental disorders in this population and an increase in the demand for care in emergency services after the first two and a half year of the pandemic, specifically due to symptoms of anxiety, depression and eating behavior [19,20]. There is also evidence of an increase in suicide attempts treated in emergency departments [15]. Therefore, there has been an increase in hospital admissions to psychiatry by 0.6% in this population, mostly due to anxiety, depression and self-harming behavior [21].

Before the pandemic, excessive screen use had already been shown to be harmful to mental health in adolescents [22]. During the pandemic period, the use of screens increased due to the need to maintain school activity at home and relationships with peers through social networks. Currently, Internet and mobile addiction are considered risk factors associated with mental disorders, mainly anxiety and depression [6,18]. Excessive Internet use reduces time spent on other beneficial activities such as physical activity, sun exposure or social relationships, and also disrupts sleep rhythms [23].

Therefore, the impact on mental health after the COVID-19 pandemic has been well documented by numerous studies, mostly of cross-sectional design using surveys. More long-term research is needed to provide more comprehensive assessment of the prevalence of mental disorders in this population following a phenomenon such as the pandemic. With the evidence that these effects continue to affect adolescents two years later, prevention and care measures must be taken for this vulnerable population group.

For these reasons, the objectives of our study were (1) to establish the association between posttraumatic stress due to COVID-19 and the presence of self-harm and suicide risk; (2) to analyze the association between stress and the presence of symptoms of anxiety and depression and eating disorder; and (3) to explore the eventual association of the presence and intensity of posttraumatic stress with the excessive use of video games and problematic Internet use.

## 2. Materials and Methods

### 2.1. Design and Procedure

The SESSAMO project (Follow-up of secondary education students to assess mental health and obesity—Seguimiento de estudiantes de secundaria para valorar salud mental y obesidad in Spanish) is a dynamic, multicenter observational cohort study of which the main aim is to assess the association between lifestyle variables and social determinants, and the mental and physical health of Spanish adolescents aged 14–16 years. The project started in December 2021, and recruitment is still open. Baseline information is collected through a digital platform. All private, public and concerted secondary schools from three Spanish regions (Navarra, Gran Canaria and Basque Country) have been invited to take part in the study. Data from participants recruited between 2021 and June 2023 from Gran Canaria (Canary Islands) and Navarra are included in this analysis (*n* = 1423).

All participants and their parents or legal guardians receive a study information sheet, as well as individual informed consents. Together with the consent of the parents/legal guardians, the assent of the minor is included. Data storage is carried out in two independent databases. One of them contains participants’ identification data and the other clinical conditions. The use of independent databases guarantees the confidentiality of the data. All information is treated in a completely confidential manner and is used exclusively for research purposes.

### 2.2. Exposure Variable: Presence and Intensity of COVID-19 Posttraumatic Stress Disorder

The presence of COVID-19 posttraumatic stress disorder (COVID-19 PTSD) was analyzed through the Brief COVID-19 Screen for Child/Adolescent (BCSCA) PTSD [24] derived from the UCLA PTSD Index, one of the most common instruments used in the evaluation of childhood and adolescent trauma. It includes an initial set of questions (e.g., *Have you or someone close to you become very sick or been in the hospital because of this illness? Has anyone close to you died because of this illness?*) to briefly review the traumatic event and set the stage for the subsequent related questions. These initial questions are followed by an 11-item set of questions about the frequency of PTSD symptoms rated from 0 = none of the time to 4 = most of the time and grouped into four symptom categories: intrusion (items 4, 7, 10), avoidance (items 1, 6), affective symptoms (items 5, 8, 9) and emotional reactivity (items 2, 3, 11). The intensity of symptoms is scored with a screener rating from 1 to 10 points: minimal; 11 to 20 points: mild symptoms; and >20 points: possible PTSD. The instrument supports the internal consistency, criterion-referenced validity and diagnostic accuracy of the screen This developmentally informed assessment tool allows researchers and clinicians to assess trauma-exposed children and adolescents (7–18 years of age) with regard to traumatic life events and DSM-5 diagnostic criteria for PTSD, including a clinical cutoff score. The reliability was 0.989 [25].

### 2.3. Outcomes

The presence of non-suicidal self-injury (NSSI) was evaluated through a specific module included in the Self-Injurious Thoughts and Behaviors Interview (SITBI) validated in Spanish [26].

Suicide risk was assessed through the Columbia Suicide Severity Rating Scale (C-SSRS) (screening version) that includes 5 items of suicidal ideation intensity (from 1 = desire to die to 5 = suicidal ideation with specific plan and intention) and a last question about suicide behaviour. The scale has been validated in Spain and is a reliable and valid instrument for assessing suicidal ideation and behaviour in research settings [27]. Suicide risk was defined when at least one of the following criteria was present: (1) Suicide behaviour plus mild/moderate ideation (1–3 point in the scale); (2) NSSI plus mild/moderate ideation (1–3 point in the scale); or (3) suicide ideation with planification and intention (4–5 points in the scale).

The presence of psychopathological symptomatology (anxiety, stress and depression) was analyzed through the Depression, Anxiety and Stress Scale (DASS-21). It has been adapted and validated to Spanish with good reliability, satisfactory convergent validity and acceptable divergent validity [28]. It is composed of 21 items. These are scored on a Likert-type scale with scores ranging from 0 = It has not happened to me to 3 = It has happened to me a lot or almost always. The three subscales of the DASS-21 each have seven items. The time frame of reference is the last 7 days. This scale ranges from 0 to 42 points for each condition. The cutoff points are: (1) for depression (normal: 0–9; mild: 10–13; moderate: 14–20; severe: 21–27; very severe: >27), (2) for anxiety (normal: 0–7; mild: 8–9; moderate: 10–14; severe: 15–19; very severe: >19) and (3) for stress (normal: 0–14; mild: 15–8; moderate: 19–25; severe: 26–33; very severe: >33).

The suspicion of the presence of an eating disorder was assessed with the Spanish version of the Children’s Eating Attitudes Test (ChEAT) [29]. This 26-item scale ranges from 0 to 78 points. Sensitivity was 27% and specificity 96%. Participants with scores higher than 20 points were considered suspected as having an eating disorder.

Internet and videogame use was assessed through two questionnaires validated for Spanish adolescents: the Scale of Problematic Internet Use in Adolescents (EUPI-a) [30] and the Game Addiction Scale for Adolescents (GASA) [31]. A participant was classified with problematic Internet use if he/she obtained a score equal to or higher than 16 points in the scale (scale range: 0–44). A participant was classified with a videogame addiction if he/she responded with a frequency of often or very often in four of the seven questions included in the scale (polythetic approach).

### 2.4. Other Covariates

Moreover, possible confounding factors were collected. Information on sociodemographic variables such as gender, school type (religious, secular, private, public), educational level of the parents, and country of birth of the adolescents and their parents was ascertained. Binge drinking and cannabis intake were also assessed. The presence of stressful life events was evaluated with the questionnaire designed by Oliva et al. [32], which consists of a list of 29 negative events, whose impact in the minor’s life is assessed. Additionally, adverse experiences such as the presence of neglect or abuse by parents or bullying was collected using validated questionnaires [33,34].

### 2.5. Statistical Analysis

The sample size was calculated assuming a difference of 5% in the proportion of any mental disorder according to extreme levels of COVID-19 posttraumatic stress disorder and with a power of 80%.

Categorial variables were described as percentages. The chi-square test for categorical variables was applied to test differences in the main characteristics of the sample according to the intensity of COVID-19 PTSD.

Logistic regression models were fitted to assess the relationship between the level of COVID-19 PTSD and the presence of several mental conditions (suicide risk or problematic Internet use or videogame addiction). Odds Ratios (ORs) and their 95% CI were calculated considering the lowest level of COVID-19 PTSD as the reference category. Moreover, COVID-19 PTSD was analyzed as a continuous variable to assess linear associations. In order to avoid the presence of confounding factors, models were adjusted for sex, mother’s and father’s academic level, mother’s country, type of school (private/concerted vs. public), impact of stressful events, presence of bullying or cyberbullying, psychological abuse by parents, parents’ negligence, sexual abuse, binge drinking and cannabis use.

Generalized linear models were used to assess the association between the presence of COVID-19 PTSD and several mental conditions such as eating disorders, anxiety and depression measured quantitatively through several screening tests. Adjusted means, regression coefficients and their 95% CIs were calculated for each test score according to the level of COVID-19 PTSD.

## 3. Results

The distribution of the main situations related to the COVID-19 pandemic among participants of the SESSAMO study are described in Table 1. Almost 10% of the SESSAMO study’s participants suffered the death of a family member during the pandemic. 

The distribution of several sociodemographic and lifestyle variables and stressful events is shown in Table 2. Participants with the highest level of COVID-19 PTSD were more likely to be females attending a public school. Moreover, the presence of bullying or cyberbullying, parents’ negligence or psychological abuse was higher in this group. Participants in the highest category also reported a higher consumption of cannabis and a binge drinking pattern.

The association between the presence of COVID-19 PTSD and several mental conditions is shown in Table 3. Participants with the highest level of COVID-19 posttraumatic stress showed an important increment in suicide risk (OR = 5.18; 95% CI = 2.96–9.05) and the presence of high scores in the ChEAT test (OR = 3.93; 95% CI = 2.21–7.00) as compared to participants with the lowest level after the adjustment for several confounding factors. Moreover, COVID-19 PTSD was directly associated with the presence of an Internet and videogame addiction. The odds of a videogame addiction were more than nine times higher in the group with the highest level of stress compared to the reference group. 

A linear association was also observed for all the mental health variables analyzed. Participants with the highest level of COVID-19 PTSD also showed the highest scores in the tests assessing eating disorders, anxiety and depression (Table 4). Multivariable adjusted mean scores for anxiety and depression observed among participants with high levels of stress were more than 10 points higher than those observed among participants with low level of COVID-19 PTSD (b coefficient = 11.1; 95% CI = 9.7–12.5 for anxiety symptoms and b coefficient = 13.0; 95% CI = 11.5–14.5 for depressive symptoms).

## 4. Discussion

The permanence of the impact of the pandemic on mental health after two years is the main result of this analysis of a sample of the adolescent population of three Spanish regions. Furthermore, female adolescents showed worse consequences in both internalizing (anxiety, depression and eating behavior) and externalizing (risk of suicide, self-harm, problematic Internet use and videogame addiction) symptoms. This greater impact on mental health in female adolescents has also been reported in other samples [7,18], although mostly associated with suffering internalizing symptoms and negative affect [35]. This trend had already been observed before the pandemic, and the multifactorial causes are not yet well understood. The main hypotheses are that men have more difficulties in recognizing mental health problems and that women react more to stressors and traumatic events such as the pandemic and its consequences [10].

Previous evidence suggests that the impact will continue in the long term, and it will depend on personal vulnerability factors and social determinants [36]. In the same way, the most negative consequences of the first waves of the pandemic had a greater impact on adolescents who had previous mental disorders [17,18], although it must be taken into account that there was a global impact on the emotional well-being of the general adolescent population [5,10,14]. The results of this study confirm the association between those who were most affected and the presence of previous events that could have made them more vulnerable. Specifically, adolescents with higher posttraumatic stress symptoms had been victims of bullying, and had suffered a greater impact from stressful life events and adverse experiences such as parental psychological abuse, parental neglect and sexual abuse. They also had higher alcohol and cannabis consumption. It is known that stressors, whether they come from peers or from adverse family conditions, leave a mark on children and adolescents that predisposes them to greater vulnerability and less effectiveness in the development of coping strategies [5].

People exposed to public health emergencies present greater psychopathological vulnerability both during and after the event [12], and the main risk factors for developing symptoms are being a woman, having had family losses and little social support, among others. In this analysis, almost a third of the participants evaluated continue to experience posttraumatic stress symptoms at a moderate–severe level two years after the pandemic, in contrast to another study that conducted a similar survey showing 71.5% of participants were affected two months after the start of quarantine [13]. The fact that 10% of the adolescents in the study experienced the death of a family member due to COVID-19 could explain, in part, this result. Although, as far as we know, no long-term research has been carried out to date, an eventual hypothesis is that vulnerable adolescents show greater emotional affectation.

In this analysis, the association between traumatic stress and the probability of developing an eating disorder is four times greater, as self-reported by adolescents. Evidence suggests that traumatic stress often precedes eating disorders. A potential mechanism could be that eating disorder behaviors serve an emotion regulation function for traumatic stress symptoms, reducing negative emotions in the short term [37]. These data confirm that this type of disorder has been exacerbated after the pandemic, especially for those who already had previous disorders [38], leading to an increase in psychiatric care and admissions for this reason [21]. Delays in health care and the loss of protective factors may explain this increase. The main risk factors that have been identified for the development of eating disorders during the pandemic are changes in eating routines, social isolation and the presence of comorbidities [17].

This health crisis has also been associated with an increase in suicide ideation and attempts and self-harming behavior in adolescents, reaching up to a 195% increase in girls [39]. This result has been related to increases in stress during the pandemic, and is also confirmed in our results, in which the participants with the highest level of posttraumatic stress showed an important increment in suicide risk. Suicidal behaviors were already a global public health problem, becoming worse with the pandemic. According to our results, the association with possible risk factors (stressful life events, family conflicts, substance use) and the increase in internalizing symptoms may have favored emotional crises over time, which has led to an increase in suicidal behavior in adolescents [40]. 

Posttraumatic stress in this population has also been associated with the presence of Internet and videogame addiction, which is nine times higher in the group with the highest level of posttraumatic stress. This could be explained as a coping strategy for perceived stress and a way of controlling stress based on the basic motivations of escape and achievement that Internet and videogames provide [41]. This finding raises the alarm that vulnerable adolescents turn to excessive use of the Internet and video games as a means of emotional regulation, distancing themselves from healthier lifestyles. 

Participants with the highest level of stress also showed the highest scores in anxiety and depression, in line with other studies that also describe these symptoms as the most prevalent [6,7,8]. Our analysis reveals the permanence of these symptoms two years after the COVID-19 pandemic, as reported by other studies in the young population [20], and it has already been noted that the psychological impact persists in the third year of the pandemic [10] without any improvement in the mental health of this population. Among the mechanisms that may explain this lack of improvement are violence in the context of family or school, decreased parental mental health during critical periods of development and changes in social behaviors like the increase in screen use [42].

This study has several limitations that may affect the generalizability of the conclusions. This is a cross-sectional study, which implies that we cannot attribute causality but exclusively associations between the impact of COVID-19 and mental health alterations. Furthermore, we do not know the mental health status prior to COVID-19 of this population sample, so an eventual reverse causality must be considered, as well as the possibility that the current consequences are due to previous poor mental health and not the other way around. Another limitation is that the information on stress is collected retrospectively, which can lead to a recall bias. This bias, on the other hand, would be a non-differential information bias that would tend to be null as no associations were found and it would be expected that the association found would still be greater and so the results were reinforced. Lastly, the procedure used in research through computerized questionnaires can lead to participants not responding accurately, thus minimizing or exaggerating behaviors that may be more stigmatized. To deal with this, one or two project technicians were present in the classroom throughout the evaluation to answer eventual questions and accompany the students when filling in the self-administered questionnaires. As strengths, it should be noted that the instruments used in this evaluation have been validated in the adolescent population. In addition, extensive information has been collected on possible factors that could distort the associations analyzed by including these variables in multivariable models.

## 5. Conclusions

In summary, two years after the pandemic there is still an impact on the mental health of adolescents. More long-term research is needed. Finally, we must highlight the importance of close monitoring and early intervention in this very vulnerable population to prevent future mental health problems.

## Figures and Tables

**Table 1:** Main situations related to COVID-19 pandemic among participants.

	**%**
Have you or someone close to you become ill or been in the hospital because of this disease?	47.2
Have you or someone close to you been quarantined for having symptoms of this disease?	77.2
Have you or anyone close to you tested positive for this disease?	80.3
Does anyone close to you work around people who might have this disease?	37.6
Have you or any of your family members had to move out of your home because of this disease?	6.1
Has anyone close to you died from this disease?	9.3

**Table 2:** Distribution of main characteristics according to intensity of COVID-19 PTSD.

	COVID-19 PTSD			
	Low (*n* = 1024)	Medium (*n* = 276)	High (*n* = 123)	*p* *
Score in COVID-19 PTSD	3.7	14.4	26.1	
Girls (%)	51.5	69.2	78.0	<0.001
Mother’s country different to Spain (%)	16.1	27.7	28.1	<0.001
Mother’s educational level (%)				<0.001
Medium	70.0	56.9	54.5	
High	16.9	26.8	21.1	
Father’s educational level (%)				<0.001
Medium	59.9	50.0	43.9	
High	20.7	30.4	26.0	
Religious school (%)	31.3	29.3	22.8	0.145
Concerted or private school (%)	35.4	29.7	22.8	0.008
Victims of bullying (%)	7.3	22.5	35.0	<0.001
Impact of stressful events (%)				<0.001
Medium	29.5	18.5	4.9	
High	25.2	28.3	26.8	
Very high	14.5	44.6	65.0	
Parents’ psychological abuse (%)	11.7	38.0	55.3	<0.001
Parents’ negligence (%)	1.7	7.6	18.7	<0.001
Sexual abuse (%)	1.8	6.5	12.2	<0.001
Binge drinking (ever) (%)	12.5	26.1	27.6	<0.001
Cannabis use (ever) (%)	3.1	8.7	16.3	<0.001

* Chi-square test.

**Table 3:** Association between the presence of COVID-19 PTSD and self-reported mental problems.

	COVID-19 PTSD			
	Low (*n* = 1024)	Medium (*n* = 276)	High (*n* = 123)	∆ 1 Point
Suicide risk				
Cases	39	63	49	
OR (95% CI)	1 (ref.)	3.59 (2.23–5.79)	5.18 (2.96–9.05)	1.09 (1.06–1.11)
Non-suicidal self-injury				
Cases	75	81	50	
OR (95% CI)	1 (ref.)	2.33 (1.56–3.48)	2.28 (1.37–3.78)	1.56 (1.03–1.08)
Eating disorder * (ChEAT test > 20)				
Cases	47	51	40	
OR (95% CI)	1 (ref.)	2.59 (1.60–4.20)	3.93 (2.21–7.00)	1.08 (1.06–1.11)
Internet addiction				
Cases	96	59	54	
OR (95% CI)	1 (ref.)	1.74 (1.17–2.60)	4.82 (2.95–7.88)	1.08 (1.05–1.10)
Videogame addiction				
Cases	14	10	10	
OR (95% CI)	1 (ref.)	2.83 (1.10–7.30)	9.49 (3.13–28.82)	1.09 (1.04–1.15)

* Analysis carried out with only female participants (*n* = 814) due to the reduced number of cases reported among boys (cases = 31). Model adjusted for: sex, mother’s and father’s academic level, mother’s country, type of school (private/concerted vs. public), impact of stressful events, bullying, parents’ psychological abuse, parents’ negligence, sexual abuse, binge drinking and cannabis use.

**Table 4:** Adjusted means for several mental scores according to intensity of COVID-19 PTSD.

	COVID-19 PTSD			
	Low (*n* = 1024)	Medium (*n* = 276)	High (*n* = 123)	*p* ANCOVA
ChEAT scores *				<0.001
Adjusted means	14.69	18.23	23.76	
(SD) (95% CI)	(1.53) (11.69–17.69)	(1.57) (15.15–21.31)	(1.66) (20.51–27.02)	
Anxiety symptoms				<0.001
Adjusted means	11.03	17.13	22.12	
(SD) (95% CI)	(0.80) (9.47–12.59)	(0.83) (15.50–18.77)	(0.90) (20.35–23.89)	
Depressive symptoms				<0.001
Adjusted means	10.44	17.23	23.43	
(SD) (95% CI)	(0.8) (8.78–12.11)	(0.89) (15.49–18.97)	(0.96) (21.54–25.32)	

SD: Standard Deviation. * Analysis carried out with only female participants (*n* = 814). Model adjusted for: sex, mother’s and father’s academic level, mother’s country, type of school (private/concerted vs. public), impact of stressful events, bullying, parents’ psychological abuse, parents’ negligence, sexual abuse, binge drinking and cannabis use.

## Data Availability

The data presented in this study are available on request from the corresponding author.
